# A Short History of the Yellow Fever Which Prevailed at Norfolk in the Months of August, September, and October, 1801; with Some Account of the Diseases That Preceded and Followed Its Appearance

**Published:** 1803-09-01

**Authors:** 


*A* SHORT HISTORY *of the* YELLOW FEVER *which prevailed at Norfolk in the Months of August, September, and October,* 1801; *with some Account of the Diseases that preceded and followed its Appearance. Communicated by* DRS. SELDEN *and* WHITEHEAD, *in a Letter to* DR. MILLER, *of New York, dated Norfolk, July* 15, 1802.

THE great influence of the weather and climate over the health of man, especially when combined, in places of crowded population, with the agency of local causes, is universally universally allowed. At no time has the truth of this observation been more conspicuously verified than during the summer and autumn of the year 1801, in most parts of Virginia, and in a particular manner at Norfolk.

The spring was extremely cold, and the progress of vegetation consequently very slow. The first week in May had elapsed before the trees were completely covered with foliage. The 25th of June had arrived before we began to feel any inconvenience from the heat of the weather, being sheltered from that degree of it, usual at this season, by frequent falls of rain, and a cloudy sky, which had hitherto prevailed. The thermometer had varied from the 70th to the 87th of Fahrenheit, which it had never exceeded, in the hottest part of the day, previous to the last six days in the month of June. But, from this time to the beginning of July, the weather was serene and intensely hot; yet no sensible effect was observed to result from it, either on the health or prevailing diseases of Norfolk, as it was immediately followed by occasional falls of rain, and that cloudy sky which had obtained so generally in the months of May and June. This state of the weather continued, with little variation, till the 23d of August.

Early in June, numbers began to be affected with intestinal fever, under the different forms of diarrhœa, or of dysentery. In most instances these complaints ran a tedious course, exhibiting symptoms of severity or mildness corresponding exactly with the variations of the weather. Nor was it in Norfolk only that febrile diseases, at this time, seemed to fall chiefly on the intestines; throughout the State similar complaints prevailed; but the attacks were more frequent, as well as more violent, in this town, Richmond and Petersburgh. The number of those affected with this form of disease increased gradually as the summer advanced; and though, in some cases, great distress was experienced, and dangerous symptoms sometimes made their appearance, yet instances of death were rare; and the greatest part were neither confined in their apartments, nor compelled to relinquish their ordinary pursuits. The town was, in other respects, during the whole summer, uncommonly healthy; and as those affected with bowel complaints were generally of the old inhabitants, who had been proof against the attack of the much-dreaded epidemic of the United States, this circumstance was regarded by some as affording reasonable grounds of belief that the atmosphere of Norfolk was free from those causes necessary to the production of yellow fever, and that, for this year, year, we should escape the ravages of this direful calamity. But these flattering hopes were speedily cut off.

During the whole of the month of August, and particularly after the 23d, some sporadic cases of yellow fever were reported to have occurred; but these were almost entirely confined to the shipping and Marine Hospital, and obtained neither the name of an epidemic, nor caused any alarm or uneasiness among the inhabitants of the town. On the 1st of September, however, the weather became extremely hot, calm, and serene. Not a cloud was to be seen, for eleven days, to afford shelter from the scorching rays of the sun, which, for that time, were poured upon us with increasing violence. By a thermometer, fixed in a passage with free circulation of air, eleven feet above the level of the street, and twelve from a door fronting the south, in a part of the town well ventilated, and not crowded with houses, the mercury frequently rose to 94, and was seldom below 90, in the hottest part of the day, from the 1st to the 11th of September. At ten o'clock in the night, during the same space of time, the mercury was found several times standing at 90 degrees of Fahrenheit.*

This extreme change of weather, in the beginning of September, was, as might have been expected, followed by one not less remarkable in the form of the diseases of Norfolk. The intestinal fever entirely disappeared, and, by the 7th or 9th of the month, the cases of yellow fever had become so numerous as to deserve the name of an epidemic, and rouse public apprehension; while those persons (as far as our observation extended) who had laboured under the former affection, were found generally not subject to an attack of the latter disease. On the 10th and 11th the number of the sick increased greatly; but, on the afternoon of the last of these days, the mercury fell to the 71st of Fahrenheit, and the wind, which had blown from the S.W. since the beginning of the month, veered about to north-

* No very accurate idea of the temperature of the atmosphere in which the inhabitants of Norfolk lived, and transacted business, can readily be collected from the thermometer, during the first three or four days after this change of weather commenced. The earth, the houses, and the air contained in them, had been so cooled by the rains and cloudy weather immediately preceding, that several days elapsed before these instruments were completely under the influence of the heat which pervaded the atmosphere of the place. To this cause is to be ascribed the very considerable variations of the mercury observed at this period in Norfolk.

north-east, with a cloudy sky, and some rain. But this change did not stop or check the progress of the epidemic: on the contrary, so much more frequent did the attacks of this form of fever become, from the 11th till the 15th of the month, that the mind was strongly impressed with the belief, that the rapid increase of disease had, in some measure, arisen from the sudden change of the weather which then took place, although such as is generally supposed favourable to health. We believe this will generally happen in situations favourable to the generation of yellow fever, when the change of temperature from heat to cold is sudden, but not sufficiently great to check, or extinguish wholly, the seeds of the disease.* Whether the cold, on this occasion, acted, according to the opinion of Dr. Rush, by accumulating excitability, and thereby rendering the system more sensible to the stimulus of the disease; or whether, by checking powerfully sensible and insensible perspiration, and detaining within the body secretions which Nature, under the influence of an unusually powerful stimulus of heat, finds necessary to eliminate, this sudden fall of the temperature overthrew the equilibrium and healthy actions of the system, in corrupting, in some measure, the whole mass of circulating fluids, and thereby disturbing and altering the various secretions formed from them.

We shall not here enter into a detail of those causes, unfriendly to health, which may be considered as arising from the situation and state of our town. Such an account would differ but little from that given in the 4th volume of the Medical Repository, p. 329, and to it we refer in this particular. But others, besides atmospherical and the local causes of Norfolk, combined to augment the spreading of the yellow fever. On account of the high price given for American produce in England, all the ships fit for the purpose that could be procured, had been constantly dispatched, during the summer, for that market; so that, in the months of July and August, the harbour was almost entirely clear of shipping, and very few sailors remained in the place. Previous to the commencement of the hot weather in September, several vessels, at different times, had arrived from Europe, with a great many passengers on board. Those who remained in town had hitherto suffered little from sickness; but, after being exposed to the extreme heat

* Vide Rush on the Yellow Fever of 1793, p. 30 et seq.

heat in the beginning of this month, many of them were taken sick; and being generally, from their indigent circumstances, badly lodged and attended, numbers of them fell victims to the disease. The wind, which, on the 11th, changed from south-west to north-east, brought in a vast number of vessels; so that, by the 15th, our harbour was crowded with ships, chiefly from Europe. The fever now spread rapidly, and increased in malignity; and many of the sailors and passengers of those newly arrived ships were among the number of the sick. It was, no doubt, unfriendly to the health of the seamen to be employed in discharging so many cargoes, near one place, at the same time. To unlock so many reservoirs of air, which had been so long pent up, and rendered impure by the circumstances of a long voyage, was not only prejudicial to the health of those who, in the presence of a prevailing epidemic, were compelled, in some measure, to live in it; but, when wafted into the adjacent parts of the town, by the wind blowing from that quarter, might contribute to augment the malignity of the disease which already existed there. About the 20th of September the disease was at its height, but it continued to the end of the month, with very little abatement of its violence; the cool weather, which came on the 27th, producing little alteration, either as to the frequency of the attacks, or the course and severity of the symptoms. It gradually declined during the month of October; and, by the 1st of November, scarce a vestige of it was to be found.

But, in proportion as the yellow fever subsided, the intestinal complaints that preceded its first attack began to re-appear. Towards the end of October, many recoveries from the fever were rendered tedious by a dysenteric affection supervening; and some who had resisted the violence of the former, sunk under the wasting influence of the latter disease. The dysentery was, however, confined to no particular class of the community, like the fever; nor was it marked with that character of malignity which often attends it when it appears as an epidemic.

The number of deaths, during the present epidemic, was fully as great, for some weeks, as in that of 1800; although, during the continuance of the fever, fewer died in this than in the former year. The deaths occasioned by the fever we are now describing, were evidently greatly augmented by the accidental arrival of vast crowds of emigrants from Great Britain and Ireland, during the violence of the fever; who, being mostly of the laborious poor, and with with large families, without the means of comfortable subsistence, were placed in the most unfavourable circumstances for recovery; and their whole families thus fell a sacrifice to the ravages of the prevailing distemper.

The plan of treatment pursued this year was, in many respects, similar to that adopted in 1800, of which we have formerly given some account. The lancet, however, was more sparingly employed, as symptoms indicating its use seemed less to require it. Calomel, in all cases, was liberally exhibited, both with a view to produce, in the commencement, a full and speedy evacuation, and afterwards, also, in such forms as have been found to bring on most readily a salivation; which, in every instance, with us, as has been often noticed by others, was followed by the certain recovery of our patient. Where topical affections occurred we had recourse to local remedies. Cupping and vesication, when early employed, afforded, in such cases, very frequently, great relief. Neither theory nor experience warranted the early exhibition of bark; we always deferred it till some change in the febrile symptoms began to appear, and the irritability of the stomach had abated: But, under every form of treatment, numbers fell victims to the disease. In this juncture, being desirous of making every effort that promised any advantage, we had recourse to a remedy we had last year tried in a few cases with some benefit, and now found attended, as far as it was carried, with unequivocal success. This was the liberal affusion of cold water; not on the plan prescribed by some of the writers of the West-Indies, but in a mode similar to that recommended by Dr. Currie, of Liverpool.

The first trials were made on young robust British seamen; and the good effects of the remedy equalled our most sanguine expectation. The pulse often, after the affusion of the cold water, was thereby reduced thirty strokes in a minute; the burning heat of the skin was greatly lessened, and the thirst, head-ach, and other uneasy symptoms, were much alleviated. The patient generally found himself so much relieved and refreshed after the cold bath, that he submitted, not only without reluctance, but with pleasure, to a repetition of it. If called in on the first or second day of the attack, we first directed a strong dose of calomel and jalap, in order to procure a full evacuation from the bowels; after which the patient was ordered to be carried on deck, with only a great coat thrown loosely around him, and three or four buckets of salt water from the river to be poured on his head and naked body. This operation was was repeated, when the febrile symptoms threatened to return with their former violence. Three times a day was usually sufficient. We rarely found it necessary to continue the use of the cold water longer than the fourth day; during which time the bowels were generally kept open by the occasional exhibition of a bolus of calomel.

From the great benefit experienced in the two or three first trials, we proceeded to recommend it with confidence. Of all those patients to whom we had an opportunity of exhibiting this remedy, on or before the second day of the attack, we had the good fortune not to lose one; but after this period, when the fever had begun to subside, without symptoms of amendment, the affusion of cold water seemed to serve only to hasten the fatal catastrophe. In no instance was it used without the exhibition of calomel at the same time; and we might have been inclined to ascribe to the last mentioned medicine the sole merit of the cure that was accomplished, had it not failed, with us, sometimes under the fairest trials.

No disagreeable effect was produced by combining the use of calomel with the affusion of cold water; nor, in a single instance, did the mercury occasion salivation, although the discharge from the bowels was scarcely as great as when it was used alone in the cure of the disease. But, in almost every instance which terminated favourably, when the cure was trusted to calomel alone, without the cold bath, some degree of salivation came on, and the appearance of this discharge was beheld with pleasure, being regarded as an infallible mark of safety.

It is with the fullest conviction of the superiority of this plan of treatment to any we have yet tried, that we venture to record its effects. The subjects of our experiments were those in whom we found the disease to attack with the greatest violence in the commencement, and to act with the most fatal force on their constitutions. We shall attempt no theory of the manner in which the salutary effects of cold bathing in yellow fever are produced, nor venture to recommend it as a certain remedy; but we think that, in the hands of a skilful and judicious physician, it may often prove a powerful auxiliary, in enabling him to combat the fatal effects of that direful calamity we have been describing.

